# Analysis of the Nanoparticle Dispersion and Its Effect on the Crystalline Microstructure in Carbon-Additivated PA12 Feedstock Material for Laser Powder Bed Fusion

**DOI:** 10.3390/ma13153312

**Published:** 2020-07-24

**Authors:** Tim Hupfeld, Alexander Sommereyns, Farbod Riahi, Carlos Doñate-Buendía, Stan Gann, Michael Schmidt, Bilal Gökce, Stephan Barcikowski

**Affiliations:** 1 Technical Chemistry I and Center for Nanointegration Duisburg-Essen (CENIDE), University of Duisburg-Essen, Universitaetsstrasse 7, 45141 Essen, Germany; tim.hupfeld@uni-due.de (T.H.); farbod.riahi@uni-due.de (F.R.); carlos.donate-buendia@uni-due.de (C.D.-B.); stan.gann@uni-due.de (S.G.); bilal.goekce@uni-due.de (B.G.); 2 Institute of Photonic Technologies (LPT), Friedrich-Alexander Universität Erlangen-Nürnberg, Konrad-Zuse-Str.3-5, 91052 Erlangen, Germany; Alexander.Blasczyk@lpt.uni-erlangen.de (A.S.); michael.schmidt@lpt.uni-erlangen.de (M.S.); 3 Erlangen Graduate School in Advanced Optical Technologies (SAOT), Friedrich-Alexander Universität Erlangen-Nürnberg, 91052 Erlangen, Germany

**Keywords:** additive manufacturing, colloidal additivation, laser fragmentation in liquids, Nanocomposites, 3D printing, selective laser sintering SLS, polyamide

## Abstract

Driven by the rapid development of additive manufacturing technologies and the trend towards mass customization, the development of new feedstock materials has become a key aspect. Additivation of the feedstock with nanoparticles is a possible route for tailoring the feedstock material to the printing process and to modify the properties of the printed parts. This study demonstrates the colloidal additivation of PA12 powder with laser-synthesized carbon nanoparticles at >95% yield, focusing on the dispersion of the nanoparticles on the polymer microparticle surface at nanoparticle loadings below 0.05 vol%. In addition to the descriptors “wt%” and “vol%”, the descriptor “surf%” is discussed for characterizing the quantity and quality of nanoparticle loading based on scanning electron microscopy. The functionalized powders are further characterized by confocal dark field scattering, differential scanning calorimetry, powder rheology measurements (avalanche angle and Hausner ratio), and regarding their processability in laser powder bed fusion (PBF-LB). We find that heterogeneous nucleation is induced even at a nanoparticle loading of just 0.005 vol%. Finally, analysis of the effect of low nanoparticle loadings on the final parts’ microstructure by polarization microscopy shows a nanoparticle loading-dependent change of the dimensions of the lamellar microstructures within the printed part.

## 1. Introduction

Additive manufacturing (AM) causes a transformation of design, manufacturing, and business models [1]. However, the range of polymer materials available for AM on an industrial scale is limited. In the field of powder bed fusion of polymers (PBF-LB/P, according to ISO/ASTM DIS 52900:2018), the market is dominated by polyamide powders with a market share of ~90% [2]. Much effort is spent on extending the range of materials in PBF-LB/P, and hereby also widen the range of potentials applications [3,4,5,6,7,8]. One approach for tailoring the polymer powder properties is additivation with nanomaterials [9,10]. Carbon-based nanomaterials, such as carbon dots [11], tubes [12,13,14], fibers [15,16] or graphene [17,18], are already known for their applicability in various fields [19,20,21] and can also be used as fillers in polymer powders for PBF-LB/P [10,22]. They significantly affect PBF-LB/P processing behavior in terms of light absorptivity adjustment for diode laser 3D printing [23,24,25,26], modify the mechanical properties [24,25,27,28,29,30], and introduce new functionalities to the printed part, e.g., electrical conductivity [23,31]. Nevertheless, the complex influence of nanofillers, related to the nanoparticle–polymer interaction during rapid heating and slow cooling of the polymer in the PBF-LB/P process, is yet not fully understood.

As nanoparticles act as nuclei during polymer crystallization and hereby strongly affect the microstructure of the printed part, it can be expected that not only the mass loading of the nanofiller, but also its dispersion (polymer surface coverage and particle size) significantly influence the powder processability and part properties. For example, high filler loadings in the range of 4 wt% of carbon nanoparticles can decrease the flexural modulus, caused by nanoparticle agglomeration [23]. On the other hand, no effects on the melt flow and the polymer crystallization were observed for loadings in the range of 0.1 wt% and low dispersion quality [30,32]. However, the degree of nanoparticle dispersion is typically not investigated in detail. One exception is a study of Meyer and Zimmerman [33], who highlighted the influence of oxide nanoparticle dispersion on the powder flowability. They found that the dispersion affects the surface roughness of the microparticles and hereby the particle adhesion, which governs the powder rheology in accordance with an earlier study by Rumpf [34]. Nevertheless, the scarce literature makes it hard to systematically correlate the processing behavior and part properties in PBF-LB to the nanoparticle dispersion. The variety of additivation methods aggravates this problem since the dispersion quality is strongly related to the nanoparticle synthesis and polymer additivation method [30,35,36], e.g., insufficient dispersion of aggregated gas phase-synthesized nanoparticles after dry coating of polymer powder or enhanced dispersion after wet coating [37].

Therefore, our study focuses on the preparation method of the feedstock material, starting with the preparation of a highly dispersed carbon colloid by laser synthesis and processing of colloids (LSPC) [38]. LSPC has become an established approach for the formation of metal and metal oxide nanoparticles [39] and was also reported by several researchers for the preparation of carbon colloids [40,41,42,43,44,45,46]. In the next step, a colloidal additivation process is used to adsorb the as-prepared laser-generated carbon nanoparticles on PA12 microparticles, directly in an aqueous dispersion. Besides a deep characterization of the nanoparticle dispersion on the polymer particle surface, we focus on investigating the heterogeneous nucleation effect caused by the nanoparticles in the polymer matrix during resolidification. Our study aims at an understanding of the influence of carbon nanoparticles especially at small nanoparticle loadings (<0.1 wt%), which are already high enough to have a high potential for influencing the polymer microstructure and the mechanical properties of the final part.

## 2. Materials and Methods

### 2.1. Colloidal Surface Additivation

**Figure 1:**
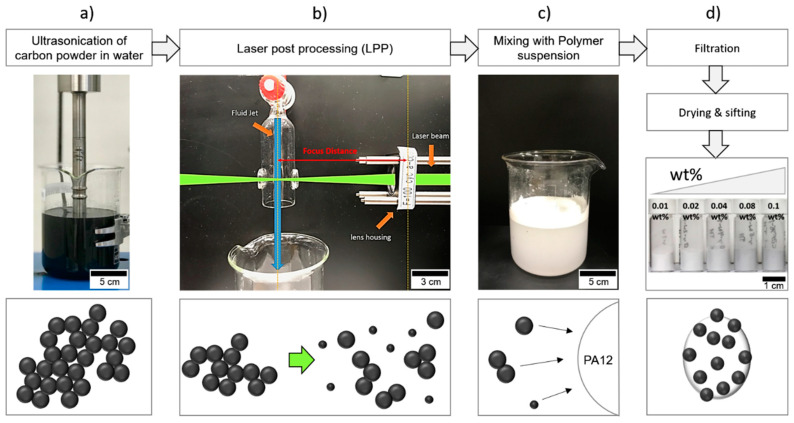
Process chain for colloidal additivation of PA12 micropowder with carbon nanoparticles: (**a**) Dispersion of aggregated carbon nanoparticle powder in water by ultrasonication. (**b**) Laser postprocessing (LPP) with a high power, high repletion rate laser, focused on the nanoparticle dispersion in a liquid jet. (**c**) Cixing of the irradiated colloid with the polymer powder and adsorption of nanoparticles on the polymer particles. (**d**) Filtration, drying, and sifting to yield dry nanofunctionalized PA12 powder.

The process chain for colloidal surface additivation of a polyamide 12 powder (EVONIK VESTOSINT 1115, Evonik Industries, Essen, Germany) is depicted in Figure 1. In a first step, 50 mg/L carbon nanoparticles (CARBON BLACK, Orion Engineered Carbons, Senningerberg, Luxemburg) is dispersed in water by ultrasonication (Hielscher, Ultrasonics, Teltow, Germany, UP200S, 200 W and 24 kHz, alternating between on and off for 0.5 s each), directly followed by laser irradiation through a cylindrical lense with a 3 ps laser system operating at 515 nm (Amphos 500flex, Herzogenrath, Germany, 5 MHz, 170 W, max. 36 mJ/cm^2^_,_ 34 µJ/pulse, 0.094 mm^2^ spot size) or with a 10 ps laser system operating at 532 nm and much higher fluence (Edgewave PX400-3-GH, Edgewave PX400-3-GH, Würselen, Germany, 80 kHz, 30 W 150 mJ/cm^2^, 375 µJ/pulse, 0.25 mm^2^ spot size). A liquid jet set-up with a flow rate of 60 mL/min and a liquid jet diameter of 1.3 mm was utilized for laser postprocessing (LPP) [47,48]. At the given spot size and repetition rate of the laser system, this leads to multiple laser pulses per volume element [49]. The applied fluence can be tuned by varying the distance between the cylindrical lens and the liquid jet so that the mass-specific energy dose can be tuned by repeating the irradiation cycle several times, which is referred to as “number of passages”. Due to self-focusing effects in the round couture of the liquid jet, approximately 12% of the liquid jet is unirradiated [50]. To ensure that >99% of the particles are illuminated, at least three passages are necessary. However, the net throughput decreases with the increasing number of passages. Therefore, only one passage was applied to the colloids in the following experiments if not stated otherwise. Hereby, 180 mg in 3.6 L of colloid can be processed per hour. After the last passage, the irradiated colloid is either analyzed via UV–Vis absorbance spectroscopy (Thermo Scientific Evolution 201, Waltham, MA, USA, 1 nm bandwidth, 0.8 nm resolution), dynamic light scattering (DLS, PSS-Nicomb 380 ZLS, Entegris, Billerica, MA, USA), or dried to perform transmission electron microscopy (TEM, Zeiss EM 910, Oberkochen, Germany), Raman spectroscopy (Renishaw InVia, Wotton-under-Edge, UK), and Fourier-transform infrared spectroscopy (FTIR, JASCO FT/IR-430, Easton, MD, USA). SEM and TEM samples were prepared by dripping 20 µL of the nanoparticle dispersion on a TEM grid or an SEM specimen mount, followed by drying for one day. To perform colloidal additivation, the colloid is directly mixed with an aqueous suspension of PA12 powder (50 g/L) under constant stirring. After irradiation, the mixture is stirred for 5 min to ensure complete supporting. The typical mass load for additivation ranged between 0.01 and 0.1 wt% (equivalent to 0.005 vol%–0.05 vol%). The concentration of 50 g/L was chosen to ensure efficient colloidal additivation. Subsequently, the mixture is filtered, dried at 50 °C for 24 h, and sifted with a 125 µm sieve before powder analysis. The educt colloids as well as the permeates after filtration were analyzed by UV–Vis absorbance spectroscopy to calculate the residual carbon nanoparticles in the permeate and the supporting efficiency, defined as

(1) $$Supporting\;efficiency=\frac{Abs_{600}\;\left(Educt\right)-Abs_{600}\;\left(Permeate\right)}{Abs_{600}\;\left(Educt\right)}100\%$$

### 2.2. Polymer Powder Analysis

The distribution of nanoparticles on the surface of the polymer particles is investigated by laser scanning confocal dark-field imaging (Leica TCS SP8, Leica Microsystems, Wetzlar, Germany) and scanning electron microscopy (SEM, ESEM Quanta 400 FEG, ThermoFisher Scientific, Waltham, MA, USA). Hausner ratio measurements were conducted according to VDI 3405 Part 1.1. The tapped volume has been determined manually with a 100 mL plastic cylinder for 5 times for statistical evaluation. The dynamic flow properties of powders were estimated by using a rotating drum analyzer (Revolution Powder Analyzer, Mercury Scientific, Newtown, CT, USA), which is known for good correlation with PBF-LB/P processing conditions due to the evaluation of particle cohesiveness during flow under elevated temperatures [8,51,52]. Avalanche angles were measured at 20 °C and 100 °C and a rotating speed of 10 rpm with a sample quantity of 100.0 ± 0.5 mL. Measurements of 150 avalanches were averaged for each sample. The dynamic image analysis (ISO 13322-2) of the PA12 powder compositions was conducted via the Camsizer X2 (Microtrac RETSCH, Haan, Germany) with compressed air of 50 kPa through the X-Jet extension to get rid of possible agglomerations. The amounts of analyzed powder per run were a few grams, which equals to around 500,000 detectable particles. The range of detection of this machine is between 0.8 µm and 8 mm, with a resolution of up to 0.8 µm/pixel. The number-weighted and volume-weighted distributions were directly calculated by the device and are based on the projected area of a sphere. The procedure has been repeated 3 times for statistical analysis.

### 2.3. Differential Scanning Calorimetry (DSC)

The PA12 powders and their composites were analyzed non-isothermally with a DSC 822e (Mettler Toledo, Columbus, OH, USA) under a nitrogen purge of 40 mL/min. The machine has a temperature accuracy of ±0.2 °C, reproducibility of ±0.1 °C, and a resolution of 0.04 µW. Powder samples of 12 mg were placed in 40 µL aluminum pans with covers. The measurements were performed from 25 °C to 250 °C with a heating rate of 20 K/min. At 80 °C, the powders were held for 3 min to ensure the same starting conditions for every powder sample. At 250 °C, the samples were held again for 3 min to fully melt all residual crystals and to assure a thermal equilibrium. Afterward, the samples were cooled down to 80 °C with a cooling rate of 10 K/min, held for 3 min, and heated up to 250 °C with 10 K/min. Thus, an analysis of the crystallization behavior and the melting behavior of previously emerged crystals as well as the sample’s crystallinity were possible. For statistical evaluation, each powder composition was analyzed 3 times, leading to a total of 9 samples. The evaluation of the results was performed with the Mettler Toledo STARe Evaluation Software 16.10. For the calculation of the relevant enthalpies, an integral tangential baseline was used.

### 2.4. Microscopic Analysis

After cooling down the DSC samples at a rate of 10 K/min, the crystalline structures were analyzed through transmitted light as well as reflected light for bright field illumination by the microscope Metalloplan from Leitz (Leica Microsystems, Wetzlar, Germany) with a magnification of 400 and 256, respectively. For this, the samples were sliced to 10 µm specimens with a microtome and embedded in oil on microscope slides. With the use of two polarizers, the birefringence of the crystals was made visible. The evaluation of the aspect ratio of the crystal forms has been performed manually by measuring the longest to shortest dimension of at least 20 crystalline structures of 3–5 DSC slices per sample, with a value of 1 being equivalent to a circle.

## 3. Results and Discussion

### 3.1. Preparation of Carbon Nanoparticles by Laser Synthesis

**Figure 2:**
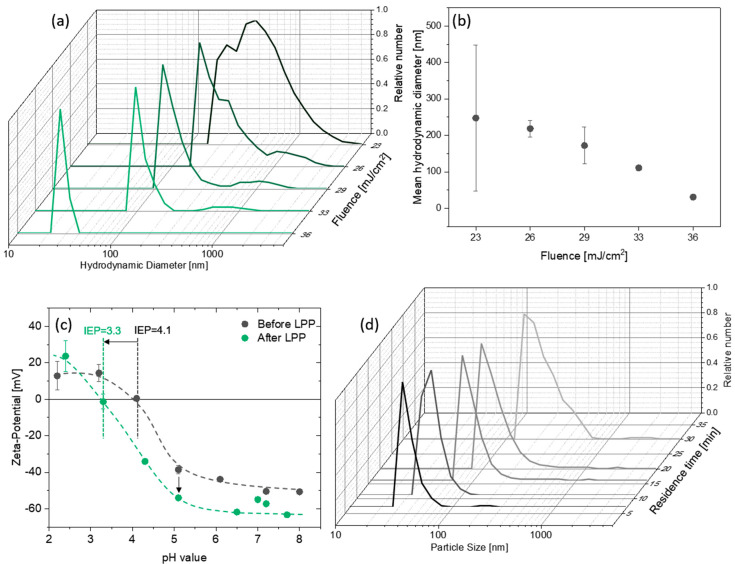
Laser postprocessing of carbon black with a high repetition rate laser system: (**a**,**b**) Hydrodynamic nanoparticle diameter (mass weighted) measured by dynamic light scattering (DLS) as a function of laser fluence. (**c**) Zeta potential as a function of the pH value of the dispersion before and after LPP. (**d**) Temporal evolution of the hydrodynamic particle size after laser irradiation. The error bars in panels (**b**,**c**) represent the standard deviation and are based on at least 3 samples each.

Ps-laser irradiation with a high repetition rate laser system (3 ps, 5 MHz, max. 36 mJ/cm^2^) significantly decreases the hydrodynamic diameter of carbon nanoparticles, which is confirmed by DLS measurements (Figure 2a). The higher the fluence, the smaller the nanoparticle size (Figure 2b). The best results were found for a fluence of 36 mJ/cm^2^, leading to a hydrodynamic particle size reduction from 157 nm to 36 nm. A similar trend can be found for the zeta potential, which decreases from −40 to −60 mV through irradiation (Figure 2c). This effect indicates a higher surface charge of the irradiated particles and has also been reported for laser irradiation of other materials, such as gold [53,54]. In accordance with the literature, the isoelectric point (IEP, Figure 2d) lies between pH values of 3.5 to 4 [55,56] and slightly shifts from 4.1 pH before LPP is to 3.3 pH through laser irradiation. Although the high absolute zeta potential value of more than 30 mV nominally indicates good colloidal stability, colloids exhibit only short time stability and show high activity for aggregation. This happens within minutes after laser irradiation (Figure 2d), indicated by hydrodynamic particles diameters in the range of a few hundreds of nm. However, the short time stability was high enough for reliable DLS and zeta potential measurements. In order to further reduce the size of the nanoparticles, another laser system with a higher fluence was utilized (10 ps, 80 kHz, 150 mJ/cm^2^). The synthesized colloid also showed very weak colloidal stability and aggregation within minutes. In both cases, the weak colloidal stability could be explained by the successful disaggregation and fragmentation of carbon nanoparticles. The smaller the nanoparticles at a given concentration, the smaller the volumetric interparticle distance, and the higher the probability for aggregation (which scales with the square root of particle number concentration).

**Figure 3:**
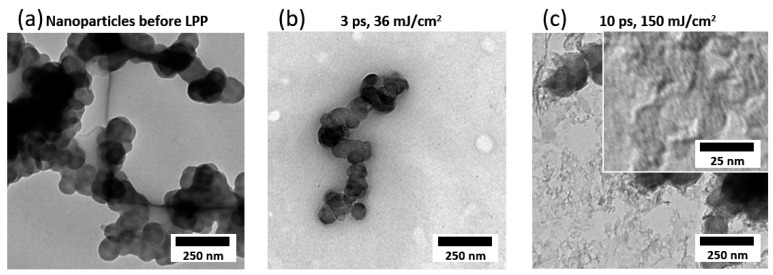
TEM analysis of (**a**) the raw carbon nanoparticles dispersed in water by ultrasonication and (**b**,**c**) colloids produced by irradiation with (**b**) a low fluence (36 mJ/cm^2^) and (**c**) a high fluence laser system (150 mJ/cm^2^). LPP with a high fluence laser system results in many small particles, but the colloid is more unstable against aggregation than at low fluence.

**Figure 4:**
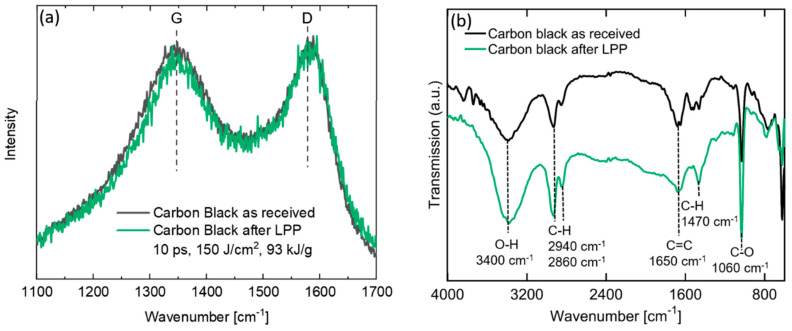
(**a**) Representative spectra from samples analyzed by Raman spectroscopy (3 measurements each), showing the two relevant peaks at 1590 cm^−1^ (G-Band) and 1350 cm^−1^ (D-Band) for carbon nanoparticles before and after irradiation with the highest fluence (10 ps, 150 mJ/cm2). (**b**) Fourier transform infrared spectroscopy (FTIR) spectra of the as-received and laser-irradiated carbon black, where the surface bonds identified by the absorption peaks are marked with a dotted line. In both cases the spectra displayed represent carbon nanoparticles before and after irradiation with the highest fluence (10 ps, 150 mJ/cm^2^).

TEM images (Figure 3) confirm pronounced disaggregation for the low fluence laser system (Figure 3b) compared to the educt particles (Figure 3a), whereas the high fluence laser system also results in fragmentation of the primary particles (Figure 3c) and appearance of strongly aggregated nanoparticles in the sub-10 nm scale (inset of Figure 3c). Laser synthesis is known to produce such small nanoparticles with a size below 10 nm [40,41,42,43,44,45,46]. Due to the high instability of the aqueous colloid and the fast aggregation after LPP, statistical evaluation of particle size and degree of dispersion based on TEM images is not meaningful as the TEM grid does not represent the degree of dispersion and the aggregate size right after laser irradiation, but the size after preparation of the TEM grid and drying of the colloid. Note that particle ripening and aggregation is much faster than drying of the colloid on the TEM grid. Despite the fraction of small particles, which is likely to dominate the number-weighted particle size distribution, a significant fraction of the educt volume material remains unchanged by laser irradiation. This is indicated by larger educt particles and aggregates on the TEM grid. In agreement with this observation, a volume-sensitive Raman shift analysis (Figure 4) does not reveal any significant changes of the ratio of the relevant peaks at 1590 cm^−1^ (G-Band) and 1350 cm^−1^ (D-Band), which result from the sp2-hybridization of planar carbon (graphite) and defect structure in the graphite, respectively. The investigation of the carbon particle’s surface by Fourier-transform infrared spectroscopy (Figure 4b) reveals the presence of C=C, C-H, C–O, and O–H bonds in the material before and after irradiation [57]. The preservation of the chemical surface groups composition after laser irradiation is associated to the employment of water as solvent, therefore no other element apart from C, H, and O are expected even after irradiation. A closer look to the spectra reveals slight differences after laser irradiation, as a higher absorption of the C–O peak at 1060 cm^−1^ and the C–H peaks at 2860 cm^−1^ and 1470 cm^−1^. This fact indicates the increased presence of C–OH and CH_2_ surface groups, which can be explained due to the higher surface area after particle size reduction and the generation of molecular O and H based radicals or molecules from water splitting during laser irradiation [58].

### 3.2. Nanoparticle Dispersion on PA12 Microparticle Surface

After irradiation of the nanoparticles, a fast aggregation of the carbon nanoparticles within minutes can be observed. However, if a support (PA12 powder) is provided under constant stirring during this phase, efficient and fast adsorption of nanoparticles on the support occurs in less than one minute. As we know from a previous study for nanoparticle adsorption on a polymer surface, constant stirring is an important factor once the colloid is instable and starts to aggregate [59]. A good mixture reduces the necessary diffusion distance between a nanoparticle and the polymer surface. The supporting efficiency of the nanoparticles on the polymer microparticles determined by UV–Vis absorbance spectroscopy of the permeate is larger than 95%, independent of the used laser for laser synthesis. Permeates show a clear color and powders exhibit a black or grey color depending on the mass load of carbon nanoparticles. As the nanoparticles completely adsorb on the polymer powder in less than one minute under constant stirring (supporting efficiency > 95%), nanoparticle adsorption is likely to dominate aggregation.

**Figure 5:**
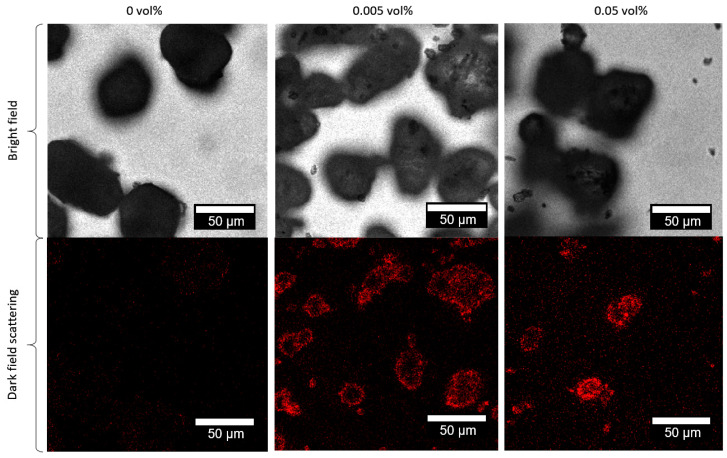
Representative confocal bright field images and laser scanning dark field images of pure PA12 and PA12 colloidal additivated with 0.005 and 0.05 vol% of laser irradiated carbon nanoparticles, respectively. For dark-field imaging, the sample was excited at a wavelength of 500 nm and the detection wavelength was set to 507–587 nm.

Laser scanning confocal microscopy of the decorated polymer particles reveals a homogeneous distribution of nanoparticles (Figure 5). It is a reference method adapted from Blaesser/Million et al. [60] and Klein et al. [61], which has also shown its potential for analyzing the distribution of fillers in polymer matrixes [62,63]. The bright-field images of the additivated and unadditivated powders look similar (Figure 5, upper row), but confocal dark-field imaging reveals much higher scattering intensity for 0.05 vol% compared to 0.005 vol%, whereas only minor scattering can be observed for the unloaded polymer powder. However, even the scattering signal from 0.005 vol% is high enough to clearly distinguish the additivated from unadditivated polymer particles.

**Figure 6:**
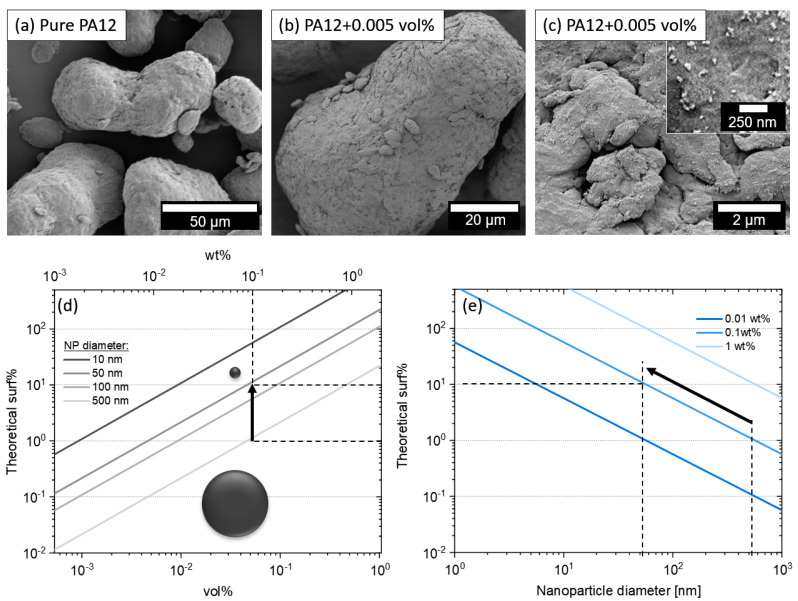
(**a**) SEM images of PA12 particles (**a**) before and (**b**) after colloidal additivation with 0.005 vol% (0.01 wt%) of carbon nanoparticles (LPP at highest fluence). (**c**) Zoom in shows the homogeneous distribution of carbon nanoparticles on the surface of the polymer microparticle. (**d**,**e**) Scaling graphs illustrating the connection between vol%, wt%, and surface coverage (surf%) for different nanoparticles sizes on PA12 polymer powder (The specific surface area of PA12 was 0.114 m^2^/g, calculated from the particle size distribution, assuming spherical particles.). The dotted lines give an example of downsizing of the nanoparticle diameter and its effect on the theoretical surface coverage.

SEM images confirm the homogeneous distribution and high dispersion of the nanoparticles on the polymer particle surface (Figure 6a–c). As expected from the colloidal analysis, small aggregates appear. The effect of dispersion and nanoparticle size on the surface coverage on the polymer particle surface is described in the scaling graphs in Figure 6d,e. Reducing the particle size by one magnitude, e.g., from 500 nm to 50 nm, also results in an increase of theoretical surface coverage by one magnitude. This is shown exemplarily in the scaling graphs in Figure 6d,e. If the particle size is dropping further to 10 nm, even 0.01 vol% of carbon nanoparticles would be enough to completely cover the polymer particles with 10 surf%. Therefore, the surface coverage could be an interesting parameter to quantify the dispersion quality and quantity, instead of just using wt% to describe the nanoparticle loading. However, for practical usage it is difficult to use this value, due to the limited resolution of SEM imaging of carbon nanoparticles on a polymer surface. This can be explained by the low contrast between nanoparticle and polymer. Especially, ultra-small carbon nanoparticles in the sub-10 nm-scale, which are generated during LPP, cannot be resolved. Eventually, the primary particle diameter cannot be determined exactly. In addition, surf% was also calculated from the polymer particle size distributions, neglecting any surface porosity of the polymer particles. Especially, the letter will highly influence a calculation of the surface coverage. However, the general scaling graphs in Figure 6d,e outline the importance of high dispersion and small nanoparticle sizes in order to reach a high surface coverage at small nanoparticle loadings.

**Figure 7:**
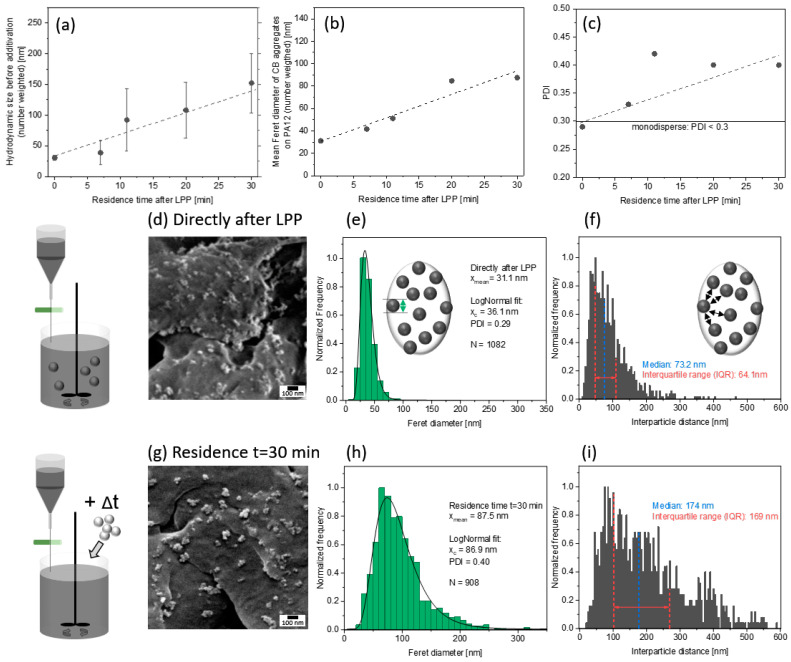
Comparison of (**a**) the hydrodynamic diameter after LPP with (**b**) the achieved Feret_max_ diameters (primary particle diameter) of carbon nanoparticles on the polymer particle surface; (**c**) the corresponding polydispersity index (PDI) of carbon nanoparticles on the polymer particle surface at 0.005 vol%. SEM image of the PA12 particle surfaces after (**d**,**g**) colloidal additivation. (**e**,**h**) Corresponding number-weighted Feret_max_ size distribution of carbon nanoparticles on the surface of PA12 particles and (**f**,**i**) interparticle distance distribution. For the comparison of particle size and interparticle distance, several images were taken for each sample at the same resolution. Nanoparticles and distances were analyzed for at ~1000 nanoparticles, e.g., N=1082 in panel (**e**) and N=908 in panel (**h**).

Although the high activity of the colloids for aggregation is a drawback for particle size analysis of the colloids, this property can be utilized to vary the dispersion of the nanoparticles on the polymer surface by variation of agglomeration after LPP. Our experimental set-up allows mixing the carbon nanoparticles with the polymer microparticles directly after laser irradiation or after a specific residence time. The hydrodynamic diameter (Figure 7a) shows a linear dependence on the waiting time between sample preparation and measurement (residence time). Comparing a direct measurement and a measurement after 30 min reveals a mean difference of more than 100 nm, which is an increase in hydrodynamic particle size of more than 200%. This reproducible effect is also reflected in the Feret diameter of the adsorbed particles and aggregates on the polymer surface (Figure 7b), and the polydispersity index (PDI; Figure 7c). The Feret diameter shifts from 25 to 80 nm, whereas the PDI increases from below 0.3 (monodisperse < 0.3) to ~0.4 after a residence time of 30 min. Please note that these measurements were just conducted once for each data point, since all SEM images needed to be analyzed manually. In addition, as mentioned before, SEM imaging of carbon nanoparticles on polymer is limited in its resolution due to low contrast between nanoparticle and polymer surface, especially on the sub-10 nm-scale. However, samples can be compared to each other, and the results in Figure 7a–c show clear tendency.

Our presented set-up allows a flexible additivation of polymer micropowders via the liquid-flow LPP and colloidal downstream supporting process. At a throughput of 180 mg of carbon nanoparticles per hour, equivalent to 3.6 L of colloid, it is capable to additivate 1.8 kg/h of polymer powder at a loading of 0.005 vol%, which is a sufficient amount for powder production to allow PBF-LB/P parameter studies and 3D printing of test structures. Equipped with a static mixer instead of a stirred tank reactor, this approach has an even higher potential for fully automated, continuous colloidal additivation.

### 3.3. PA12 Powder Characteristics

**Figure 8:**
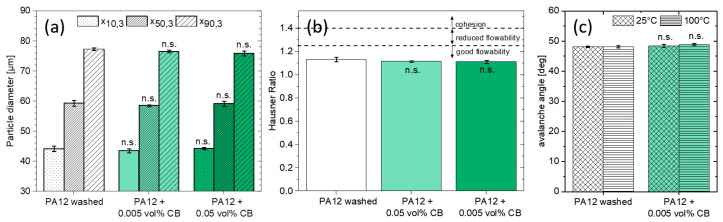
(**a**) Polymer microparticle size distribution, (**b**) Hausner ratio, and (**c**) avalanche angle before and after colloidal additivation for 3 samples each. Particle sizes are based on the projected area of a sphere x_area_. The significance analysis shows no significant differences (n.s.) through the addition of different amounts of CB.

To ensure the processability of the nanoparticle–polymer composite powder in terms of powder recoating/spreading during PBF-LB/P, colloidal additivation should not worsen the morphology and geometry of the base particles, preserving the extraordinary flowability of the base powder material. Therefore, particle size distribution before and after colloidal additivation is analyzed. The addition of 0.05 and 0.005 vol% carbon nanoparticles (CB) does not show significant differences (n.s.; P>0.05) in the x_10,3_, x_50,3_ and x_90,3_ of volume-weighted distribution (Figure 8a). Furthermore, all particle size distributions are located within the desired average particle size of 10 and 120 µm for PBF-LB/P [64]. Minor changes of particle size distribution are only significant for the number-weighted distribution. However, the total impact is rather minimal and should not negatively influence the flowability of the powders. This hypothesis could be validated by measuring the Hausner ratio (HR). Here, the additivation of small amounts of CB does not significantly (n.s.; P>0.05) affect the good flowability (<1.25) of the base powder (Figure 8b), which shows a Hausner ratio similar to typical PA12 powders used for PBF-LB/P [65]. This is also confirmed by dynamic flow properties, characterized by the unchanged avalanche angle (Figure 8c).

### 3.4. Analysis of the Crystallization Behavior

**Figure 9:**
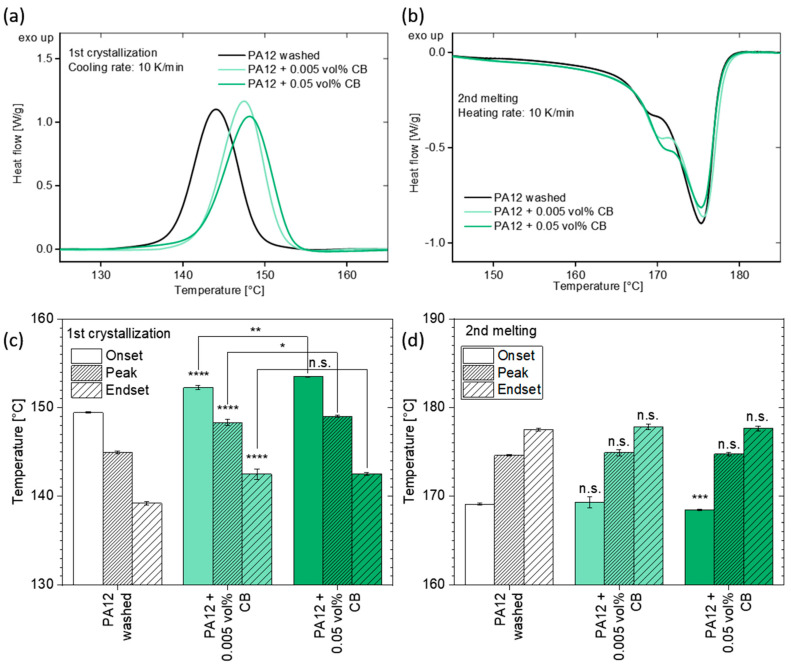
DSC analysis of PA12 powder before and after colloidal additivation with the laser-generated carbon nanoparticles: (**a**) 1st crystallization and (**b**) 2nd heating curves, based on the average of 3 runs. (**c**,**d**) Extracted onset, peak, and endset temperatures with corresponding error bars. The analysis of statistical significance either shows no significant difference (n.s.; *p* > 0.05) or a significant difference, where the *p*-values decrease with the increasing number of asterisks.

Particles of nanoscale dimensions can significantly influence the melting enthalpy and act as heterogeneous nucleation seeds, increasing the crystallization temperature and initiating crystal growth during cooling [66,67,68]. Even small amounts (0.005 vol%) of CB induce a change of the crystallization, and subsequent melting behavior as shown in the DSC analysis (Figure 9a,b). The crystallization onset, peak, and endset temperatures shift significantly (****; P≤0.0001) to 3 °C higher temperature values (Figure 9c). The addition of 0.05 vol% CB increases the onset and the peak temperature even further (+5 °C) compared to the base material. Based on these thermal results, CB seem to act as heterogeneous nucleation seeds already at a minute amount of nanoadditive. By subsequently heating the DSC samples, the melting behavior of the crystalline structures can be analyzed (Figure 9b,d).

**Figure 10:**
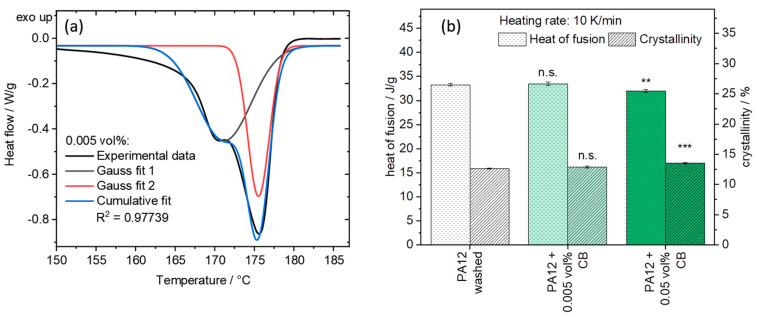
(**a**) Exemplary determination of the heat of fusion of both peak areas from the DSC second heating curve and (**b**) heat of fusion and crystallinity of PA12 and its composites after colloidal additivation calculated from the DSC data. No significant changes are visualized with n.s. (*p* > 0.05) and significant differences with asterisks ** (*p* ≤ 0.01) and *** (*p* ≤ 0.001).

The addition of CB leads to an increase in the heat of fusion $\Delta H_{m}$ at ~172 °C (Figure 10), displayed by a larger peak area (Figure 10b), which indicates an increasing number of thinner lamellar crystalline structures [69]. From the heat of fusion, the crystallinity $X_{c}$ of the samples was calculated according to Equation (2) [70,71]:

(2) $$X_{c}=\frac{\Delta H_{m}}{\Delta H_{100}\cdot \left(1-w_{f}\right)}=\frac{\Delta H_{m}}{209.3\;\frac{J}{g}\cdot \left(1-w_{f}\right)}$$

where the enthalpy of fusion of a 100% crystalline PA12 crystal $\Delta H_{100}$ is given in the literature [72], and $w_{f}$ is the weight percentage of the nanofiller in the composite. At higher amounts of CB (0.05 vol%), the overall heat of fusion shows a significant (**; *p* ≤ 0.01) decrease compared to the base material, while the crystallinity increases significantly (***; *p* ≤ 0.001) (Figure 10b). Based on these measurements, higher amounts of CB seem to promote the crystal growth at a cooling rate of 10 K/min. This correlates with the fact that more CB lead to more nucleation sites, increasing the amount of crystalline structures. 

**Figure 11:**
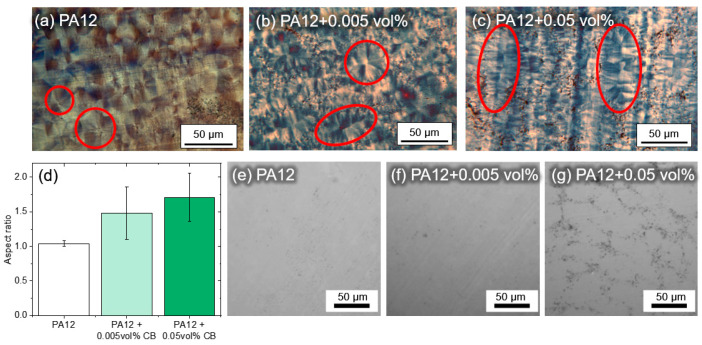
(**a**–**c**) Polarization microscopy images of PA12 with different carbon nanoparticles loading, showing representative crystalline structures of different dimensions. (**d**) Corresponding aspect ratio of observed crystalline shapes at different carbon nanoparticles loadings. (**e**–**g**) Bright-field images of PA12 with carbon nanoparticles, depicted as black dots. Samples were analyzed after sizing to 10 µm films.

For a more detailed analysis of the nucleation effects of CB, an evaluation of the area underneath the two peaks of the heat of fusion curve of Figure 9b was conducted in Figure 10a. The deconvolution of two Gaussian curves (R^2^ > 0.97) shows that the left area increases with increasing amounts of CB from 18.10 to 21.38 and finally to 22.57 J/g, while the right area sank from 16.18 to 13.57 and finally to 11.01 J/g, accordingly. This leads to the conclusion that the addition of CB increases the amount of thinner lamellar crystals while decreasing the amount of thicker crystalline structures [69]. Further proof for different crystal dimensions can come from polarization microscopy imaging of sliced DSC samples. Pure PA12 samples show the typical spherulite structures of a Maltese cross (Figure 11a) with an aspect ratio of 1.04 ± 0.04. The origin of these crystals is located in their center. The addition of 0.005 vol% CB leads to different crystalline structures instead. Some of the typical round spherulite structures are replaced by oval shapes at 0.005 vol% CB (Figure 11b) with an aspect ratio of 1.48 ± 0.38. This phenomenon becomes further evident when increasing the amount of CB to 0.05 vol% and thereby the density of the carbon nanoparticles on the polymer matrix. In this case, oval structures dominate the crystalline areas in the sample (Figure 11c), resulting in an even higher aspect ratio of 1.71 ± 0.35 (Figure 11d). The transition point between solely spherulites and only oval lamellar structures seems to lie between 0.005 and 0.05 vol% CB in PA12.

**Figure 12:**
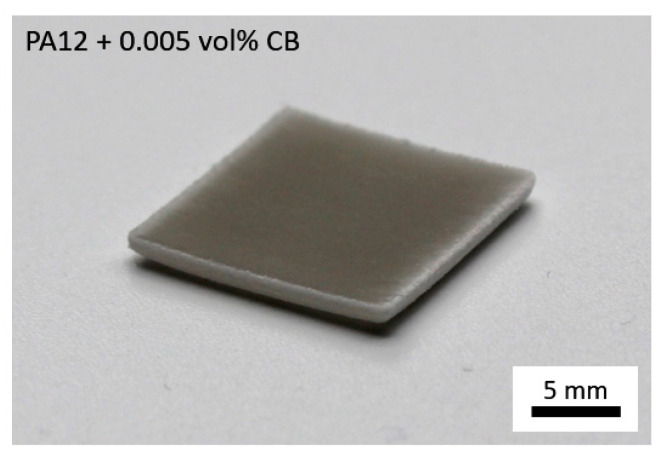
Test sample generated by PBF-LB/P of PA12 powder additivated with 0.005 vol% carbon nanoparticles.

An explanation for the change of crystal structure could lie in the nanoparticle dispersion within the polymer matrix. Incident bright-field images of the sample slices are shown in Figure 11e–g. Increasing the amount of CB to 0.05 vol% clearly shows the CB distribution throughout the polymer matrix. The CB, which were initially adhered to the surface of the powder particles, are distributed on former polymer powder particle surface, creating a superstructure on the former surface of the polymer particles, visible as chain formation in the sliced samples. As a result, spherulites which originate on these nucleation seeds show highly anisotropic growth. This transition in crystal structures could have an effect on mechanical properties of final parts and should be examined in future studies. To prove that PA12 powders additivated with 0.005 vol% of colloidal nanoparticles can be processed on a PBF-LB/P machine, a test sample of 10 layers was successfully printed on an EOSINT P385 (150 µm layers, 13 × 13 mm^2^) (Figure 12), indicating high potential for generating test structures of refined parameters for an optimal layer bonding in follow-up studies on the influence of a very low carbon nanoparticle dose on the microstructure and properties of printed polymer parts.

## 4. Conclusions

Carbon nanoadditives are often applied at high weight doses in polymer feedstock powder for PBF-LB/P. This limits the dispersion and decreases the number of nuclei for heterogeneous nucleation of the polymer. Alternatively to chemical ligands as dispersion aids, which might hinder the polymer–nanoparticle bonding significantly, lower additive doses could be applied in case a good dispersion on the polymer surface is achieved. Hence, the influence of the degree of nanoparticle dispersion is an important aspect for the application of nanofunctionalized polymer powders in PBF-LB/P. By colloidal additivation of PA12 with carbon nanoparticles in an aqueous dispersion at comparable small loadings, we were able to coat the polymer particles with a homogenous layer of carbon nanoparticles. Colloidal nanoparticles were dispersed and fragmented in water by laser postprocessing (LPP), prior to colloidal additivation. Our experiments reveal that the dispersion of the carbon nanoparticles on the polymer surface can be tailored by the waiting time between sample preparation and measurement (residence time). If direct mixing was performed immediately after laser irradiation, the nanofunctionalized PA12 shows a high carbon nanoparticle dispersion on its surface with a polydispersity index PDI < 0.3.

As-prepared feedstock materials trigger heterogeneous nucleation effects even at just 0.005 vol% of carbon nanoparticles, underlining the value of high dispersion. Through their high surface coverage (surf%) and their small interparticle distances of 50–100 nm, the carbon nanoparticles form a superstructure after melting the polymer matrix. Hereby, they affect the lamellar dimensions of the crystalline structures. The form of the crystals changes from typical round spherulites to oval lamellar structures with the addition of more carbon nanoparticles. Exemplary PBF-LB/P experiments show that the modified PA12 powder with 0.005 vol% CB can be processed like the raw PA12 powder. A deeper understanding of nanoparticle influence on polymer crystallization at small nanoparticle doses will facilitate a precise modification of the microstructure and could have significant effects on the mechanical properties of printed parts.
